# Emerging concepts in cancer therapy: Mechanisms of resistance

**DOI:** 10.1002/cnr2.1715

**Published:** 2022-09-09

**Authors:** Seema Gupta, Vivek Verma, Bilikere S. Dwarakanath

**Affiliations:** ^1^ The Loop Immuno‐Oncology Laboratory, Lombardi Comprehensive Cancer Center Georgetown University Medical Center Washington District of Columbia USA; ^2^ The Hormel Institute, Masonic Cancer Center University of Minnesota Austin Minnesota USA; ^3^ Central Research Facility Sri Ramachandra Institute of Higher Education and Research Institute Chennai India

Inherent and induced resistance offered by tumors to various therapeutic modalities is a major impediment in achieving success in anticancer therapies.^1^, ^2^ Targeted therapies such as small molecular inhibitors and their combination with other treatments such as immunotherapies showed promise initially.^3^, ^4^ However, unfortunately, owing to primary and secondary mechanisms, tumors develop resistance against combinational, targeted and immune therapies that remain a formidable challenge to the experimental and clinical oncology community. This issue of Cancer Reports has put together commentaries, reviews, and original contributions covering the current understanding of some of the aspects of therapeutic resistance in contemporary cancer therapy.

C. Pathak and colleagues provide a brief review on the interplay between diverse intracellular signaling pathways as well as the interactive processes between various cellular components in the tumor microenvironment that contribute to the multidrug resistance (MDR) against most arms of the cancer therapy.^5^ They conclude that obtaining a deeper understanding of the molecular mechanisms that contribute to the MDR and therapy‐induced functional reprogramming of tumor cells is a major challenge.^5^ Therefore, to improve the efficacy of anticancer therapies, prospective efforts should focus on this important area, which would provide better opportunities to design newer therapies to overcome drug resistance.

Establishing a vincristine–cisplatin resistant human carcinoma cells (KB), A. Sridhar and colleagues showed that a cross talk between matrix metalloprotease 7 (MMP 7) and heat shock protein 90 (HSP90) could facilitate tumor progression and metastatic potential besides leading to acquired drug resistance.^6^ They also demonstrated that enhanced homing in MMP7 overexpressing tumor cells could be inhibited using the HSP90 inhibitor 17‐allylamino‐17‐demethoxy‐geldanamycin (17‐AAG).^6^ Since MMP7 inhibitors are already undergoing clinical trials, while HSP90 inhibitors are awaiting approval, their findings provide a novel approach for overcoming drug resistance by targeting simultaneously MMP7 and HSP90.

For several decades, induction of angiogenesis in tumors has been considered as one of the hallmarks of cancer.^7^, ^8^ Accordingly, several antiangiogenic drugs, including bevacizumab, have been employed as anticancer agents. However, resistance to these therapies has been observed in both animal tumor models and patients. Notably, discovery of nonangiogenic tumors suggested that mechanisms other than neoangiogenesis exist, leading to development and metastasis of such tumors.^9^ Based on preclinical and clinical studies, F. Pezzella and D. Ribatti describe two mechanisms of intrinsic and acquired resistance to antivascular agents: vascular co‐option and vasculogenic mimicry.^10^ Identification of these mechanisms is promising as it provides novel targets for anticancer therapies, especially vascular co‐option being an active process that involves specific pathways such as cell motility and integrin signaling pathways that may potentially be targeted alone or in combination with other therapeutic strategies.

Radiation therapy (RT) has remained one of the long‐standing treatments for cancer. However, RT can also induce pro‐survival factors mediated by multiple signaling pathways, such as AKT, ERK, and ATM/ATR.^11^ Induction of pro‐survival molecules through these pathways suppress cell death, induce cell cycle arrest, and facilitate DNA repair, leading to radioresistance. Using human glioma cells, B.S. Dwarakanath and coworkers presented evidence suggesting that the radioresistance of cancer cells could be linked to the levels of calreticulin, an endoplasmic reticulum (ER) resident protein involved in maintaining calcium homeostasis in ER.^12^ Calreticulin is also implicated in the immunogenic cell death. Moreover, they also showed that the calreticulin linked radioresistance can be overcome by administration of polyphenolic acetate 7,8‐diacetoxy‐4‐methylcoumarin, an acetyl group donor to a calreticulin‐mediated acetylation, thus altering the damage response pathways induced by radiation.^12^ In another study, S. Gupta and M.M. Ahmed showed that ionizing radiation induces binding activity and expression of upstream stimulatory factor‐1 (USF‐1) in prostate cancer cells.^13^ They demonstrate that upregulation of USF‐1 serves as a marker of radioresistance since the expression of target genes of USF‐1 that are involved in proliferation and survival are increased in cancer cells having overexpressed USF‐1 following irradiation. Like most transcription factors, binding of USF‐1 to the promoters and its ability to cooperate with other factors involved in establishment of an active transcription complex can be modulated by acetylation/deacetylation events. Therefore, the authors investigated the effects of histone deacetylase (HDAC) inhibitors on functions of USF‐1 and found that HDAC inhibitors reduce the expression of radiation‐induced USF‐1 and its targets.^13^ Additionally, HDAC inhibitors reduced the proliferation of USF‐1‐overexpressing cancer cells compared with radiation, indicating that reversal of radioresistance by HDAC inhibitors may be mediated by inhibition of USF‐1.^13^ The findings from these two studies will help in the development of novel strategies to overcome radioresistance of cancer cells.

Since the heterogeneity of tumor response to various anticancer treatments is now well established, there is an increased interest in identifying the predictive biomarkers of response or resistance. This will not only lead to the design of personalized treatment but also in the development of novel strategies to enhance antitumor response and reverse resistance. T.A. Theodossiou and coauthors in an elegant study established crucial biomarkers specific for 5‐aminolevulinic acid (5‐ALA)‐photodynamic therapy (PDT).^14^ They correlated the differential cytotoxicities of two human glioblastoma (GBM) cell lines and three breast adenocarcinoma cell lines to 5‐ALA‐PDT with their inherent characteristics. Using multiple inhibitors that either affected the intracellular accumulation of protoporphyrin IX (PpIX) (ABCG2 transporter), or production of PpIX and its conversion into heme (heme oxygenase, HO‐1; ferrochelatase, FECH), or inherent antioxidant cell defenses (glutathione synthetase), they identified the critical factors affecting 5‐ALA‐PDT‐induced cell death.^14^ Since these factors may potentially serve as specific biomarkers predicting the outcome of 5‐ALA‐PDT, the authors suggest that based on the analysis of tumor biopsies, personalized 5‐ALA‐PDT treatments and their combinations can be designed.

Triple negative breast cancer (TNBC) is a leading cause of death in women worldwide. Besides primary disease, metastasis of TNBCs is associated with worst prognosis.^15^ D. Raman and A.K. Tiwari have provided a commentary on the underlying mechanisms of metastasis and disease progression with special reference to cancer stem cells and their role in disease recurrence.^16^ They identified the role of eukaryotic translation initiation factor 4A1 (eIF4A1) in cancer cell stemness and in regulating translation of oncoproteins such as survivin, cyclins, Rho kinase 1, and ADP ribosylation factor 6. These eIF4A1‐regulated proteins are vital for tumor cell survival, proliferation, migration, invasion, and chemoresistance. Besides, eIF4A1 also regulates the expression of drug transporters on tumor cell surface, thus contributing to chemoresistance. Hence, the authors emphasize the importance and potential of targeting eIF4A1 in preventing TNBC metastasis and reversing the resistance to therapy.^16^

Recently, metabolism has emerged as a crucial factor in deciding the disease progression and the response to therapeutic interventions.^17^ Interestingly, the effect of metabolism in mothers has been identified to influence the risk of breast cancer generation in female offspring.^18^ Here, F.O. Andrade and coauthors have provided a review on the impact of maternal obesity on the tendency of female offspring to generate breast cancer. They explored the previously unappreciated link between gut dysbiosis in the mothers and its influence on disease progression and resistance to therapeutic interventions in the offspring. With a special emphasis on metabolites such as short‐chain fatty acids (SCFAs) produced by the microbiota, the authors discuss how these metabolites influence the immune system by modulating the population and function of immune cells such as regulatory and effector T cells. They also discussed the impact of microbiota‐driven factors such as SCFAs and lipopolysaccharides on modulating the immune system and hence the response to immune‐based antitumor therapies.

Multiple approaches that are described in this issue and many others that are either under clinical evaluation or being developed are expected to improve the efficacy of cancer therapy by overcoming the resistance offered by tumors to various mono and combined therapeutic modalities. We are grateful to all the authors for timely and invaluable contributions to this special issue. We thank the Editor‐in‐Chief of Cancer Reports for the kind invitation to edit this issue and the editorial staff of the journal for their unstinted help in putting this issue together.
